# Revision TKA with a distal femoral replacement is at high risk of reinfection after two-stage exchange for periprosthetic knee joint infection

**DOI:** 10.1007/s00167-021-06474-2

**Published:** 2021-02-10

**Authors:** Christoph Theil, Kristian Nikolaus Schneider, Georg Gosheger, Tom Schmidt-Braekling, Thomas Ackmann, Ralf Dieckmann, Adrien Frommer, Sebastian Klingebiel, Jan Schwarze, Burkhard Moellenbeck

**Affiliations:** Department of General Orthopedics and Tumor Orthopedics, Muenster University Hospital, Albert-Schweitzer-Campus 1, 48149 Muenster, Germany

**Keywords:** Periprosthetic joint infection, PJI, Revision total knee arthroplasty, Revision TKA, Megaprosthesis, Megaprostheses

## Abstract

**Purpose:**

Megaprosthetic distal femoral reconstruction (DFR) is a limb-salvage procedure to address bone loss following two-stage revision for periprosthetic knee joint infection (PJI). The purpose of this study was to analyze the survival of DFR compared to hinged total knee arthroplasty (TKA). It was hypothesized that DFR was associated with a poorer survival.

**Methods:**

In this retrospective single-center study, 97 subjects who underwent two-stage revision of chronic knee PJI were included. Among these, 41 were DFR. The diagnosis of PJI was established using the Musculoskeletal Infection Society (MSIS) criteria. Implant survival was calculated using Kaplan–Meier method and compared with the log-rank test as well as multivariate Cox regression at a minimum follow-up period of 24 months.

**Results:**

The median follow-up period was 59 (interquartile range (IQR) 45–78) months. Overall, 24% (23/97) of patients required revision surgery for infection. The infection-free survival of rotating hinge revision TKA was 93% (95% Confidence Interval (CI) 86–100%) at five years compared to 50% (95% CI 34–66%) for DFR. In multivariate analysis, the risk factors for reinfection were DFR reconstruction (HR 4.7 (95% CI 1–22), *p* = 0.048), length of megaprosthesis (HR 1.006 (95% CI 1.001–1.012), *p* = 0.032) and higher BMI (HR 1.066, 95% CI 1.018–1.116), *p* = 0.007). 10% (4/41) of patients undergoing DFR underwent amputation to treat recurrent infection.

**Conclusion:**

Megaprosthetic DFR as part of a two-stage exchange for PJI is a salvage treatment that has a high risk for reinfection compared to non-megaprosthetic TKA. Patients must therefore be counseled accordingly.

**Level of evidence:**

Retrospective observational study, Level IV.

## Introduction

Periprosthetic joint infection is one of the most devastating complications following total knee arthroplasty (TKA) and the prevalence is estimated to be about 1–2% for primary joint replacements [17]. Frequently a two-stage revision that consists of removal of the implant, thorough debridement and insertion of a polymethylmethacrylate (PMMA) spacer loaded with antibiotics is performed combined with systemic antibiotic treatment [11, 23]. When the microorganisms are considered to be eradicated, prosthesis reimplantation is performed. However, there is still a risk of reinfection in up to 20% [4]. Particularly in repeated revision surgeries for infection, failures are commonly observed [9].

One of the most challenging problems following debridement of infected tissue is bone loss and can result in a large metaphyseal bone defect with loss of the femoral condyles that can even extent to the diaphysis [9, 14]. For such defects, modular megaprosthetic reconstruction, of the distal femur can be considered [2, 14, 25] based on the long-term experience from tumor surgery [12, 13, 24]. However, due to the expected high revision rates, these procedures should be considered as salvage treatments [1, 14]. The mid- to long-term revision free survival of non-oncological megaprosthetic distal femoral reconstruction has been reported to be around 50% with (re-) infection being one of the main complications [29]. Previous studies on distal femoral megaprostheses for non-oncological indications focused mainly on periprosthetic fractures [29]. Thus, only little is known about complications of such megaprostheses when used for knee PJI [26].

Thus, the aim of the present study is to investigate the eradication of infection in knee PJI comparing megaprosthetic distal femoral and non-megaprosthetic reconstruction, the infection-free survival of these implants and risk factors for failure. It was hypothesized, that megaprosthetic distal femoral reconstruction is associated with an increased risk for reinfection as compared to rotating hinge TKA for the treatment of PJI.

## Patients and methods

This study was approved by the local ethical committee (reference number 2019-650-F-s) and was conducted in accordance with the principles established by the world medical association of Helsinki.

**Fig. 1:**
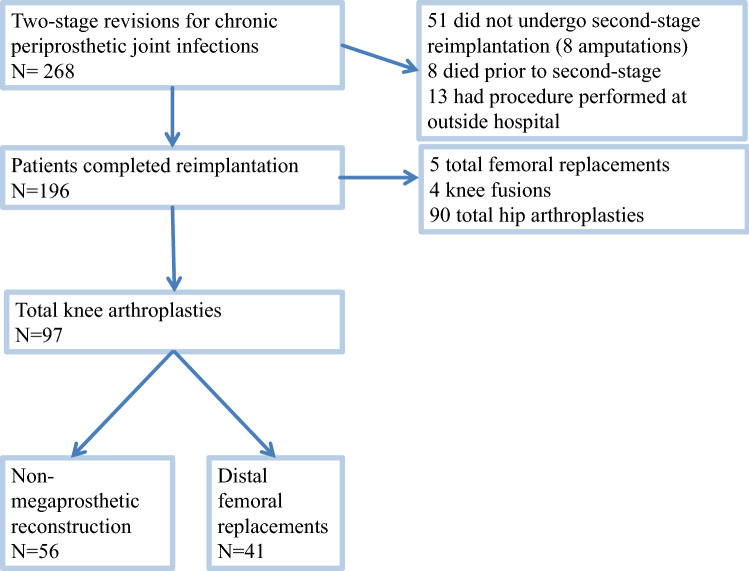
Flow chart showing patient inclusion and distribution for revision TKA in the present study

A retrospective database research of the institutional arthroplasty registry was conducted. In summary, 97 subjects who underwent staged revision and implant exchange for chronically infected TKA between January 2012 and December 2016 were identified and included for final analysis. Megaprostheses for oncological reconstructions as well as four megaprosthetic knee fusions for PJI (Fig. 1) have been excluded. In total, 56 non-megaprosthetic revision TKAs and 41 distal femoral replacement revision TKA were available for analysis. Patient’s previous medical history was analyzed to calculate the Charlson Comorbidity Index (CCI) [3], body mass index (BMI) and previous surgical history with respect to previous infections and revision surgeries (Tables 1, 2).

**Table 1:** patient demographics and surgical details

Variable	DFR % (*n*)	Non-mega-prosthetic % (*n*)	*p* value
Male	49 (20/41)	45 (25/56)	0.837
Diabetic	29 (12/41)	30 (17/56)	0.545
Previous prosthesis revision surgery	76 (31/41)	54 (30/56)	0.034
Previous revision for PJI	54 (22/41)	30 (17/56)	0.023
Previous aseptic prosthesis revision	42 (17/41)	32 (18/56)	0.395
Culture negative infection	22 (9/41)	30 (17/56)	0.348
Polymicrobial infection	7 (3/41)	13 (7/56)	0.511
Mortality	16 (11/41)	26 (9/56)	0.214

All *p* values > 0.05 should be n.s

**Table 2:** patients’ demographics and surgical details

Variable	DFR Median (25–75% interquartile range)	Non-mega-prosthetic Median (25–75% interquartile range)	*p* value
Age at surgery in years	73 (63–77)	68 (59–76)	0.07
BMI in kg/m^2^	31 (28–36)	29 (26–36)	0.293
Number of previous revisions			
Septic revisions	1 (0–3)	0 (0–1)	0.003
Aseptic revisions	0 (0–1)	0 (0–1)	0.396
Follow-up period in months	54 (36–62)	65 (49–83)	0.004
Charlson comorbidity index	3 (2–5)	2 (0–4)	0.160
Femoral reconstruction in mm for DFR	120 (90–170)	None	N/A

All *p* values > 0.05 should be n.s

The diagnosis of PJI was made based on the musculoskeletal infection society (MSIS) as published in 2011 [22]. Specifically, serum C-reactive protein (CRP), synovial leukocyte count and percentage of neutrophils, as well as interleukin 6 (IL6), were used to guide the decision-making. Furthermore, clinical findings, such as pus and fistula, were considered as well as microbiological findings from synovial fluid cultures or intraoperative tissue samples. Three to five microbiological samples were taken from different macroscopically affected areas of the joint intraoperatively. The cultures were grown for a minimum of 10–14 days on Schaedler agar, chocolate agar and Columbia blood agar. Prior to reimplantation, all wounds must have healed and a clear improvement of serum inflammatory markers must have been evident. Local antibiotics were delivered with a static spacer design using handmade polymethylmethacrylate (PMMA) spacers that were stabilized with intramedullary rods with gentamicin and clindamycin added for sensitive organisms. Vancomycin was added for Gram-positive bacteria and Meropenem for Gram-negative organisms, as well as amphotericin for fungal infections. A tailored intravenous and oral systemic antibiotic therapy of at least six weeks was administered. Following re-implantation, the same antibiotic regimen from first-stage surgery was administered until final cultures had returned negative and undisturbed wound healing was present. If cultures from the reimplantation surgery were positive, oral antibiotics were continued for another four weeks. The primary endpoint was further revision surgery for infection. Eradication of the infection was defined as stated by Diaz-Ledema et al. requiring healed wounds, no further revision surgery for infection and no PJI related mortality [6].

The size of the bone defect was assessed using preoperative anterior–posterior and lateral radiographs as well as intraoperative fluoroscopic imaging. Based on these findings, the defect was classified according to the Anderson Orthopedic research institute (AORI) classification (Table 3) [7].

**Table 3:** defect classification on the femoral and tibial side according to the Anderson Orthopedic Research Institute classification

Classification of femoral and tibial bone defects	DFR % (*n*)	Non-mega-prosthetic % (*n*)
Femur		
1	0	50 (28/56)
2	0	48 (27/56)
≥ 3	100 (41/41)	2 (1/56)
Tibia		
1	42 (17/41)	63 (35/56)
2	51 (21/41)	34 (19/56)
3	7 (3/41)	4 (2/56)

The indications for megaprosthetic distal femoral reconstruction included femoral type 3 defects or even larger defects with complete loss of the distal femur. Smaller defects were reconstructed using modular metal wedges and bone cement aiming for a stable metaphyseal and diaphyseal reconstruction. Bone grafting for defect reconstruction was not performed. The MUTARS-system (modular universal tumor and revision system, Implantcast, Buxtehude, Germany) was used for all reconstructions with the GenuX system being used for all non-megaprosthetic rotating hinge knee reconstructions. This system uses a rotating hinge design and offers modularity allowing for reconstruction of the joint line with metal wedges on the tibial and femoral side, as well as segmental modular megaprosthetic reconstruction of the bone defect to the nearest centimeter. All non-megaprosthetic components were fixed using a hybrid fixation (uncemented stem and cemented tibial plateau) or full cementation using PMMA cement (Copal G + C or G + V, Heraeus Medical, Wehrheim, Germany). The choice to perform a cemented or uncemented stem fixation was based on bone quality, previous implant fixation, desired metaphyseal or diaphyseal fixation and the diameter of the bone. For DFR, an uncemented fluted stem was used in 30 cases and was combined with a fully cemented, stemmed tibial component in 8 cases, otherwise tibial hybrid fixation was chosen. In the remaining 11 cases, a non-fluted femoral stem was used that was cemented in six cases. All of these cases were combined with a hybrid-fixated tibia. For non-megaprosthetic reconstruction, 48 revision TKA were implanted with a hybrid fixation technique while 8 implants were fully cemented.

Patients were followed clinically and radiographically at 3–12 months and then annually. Follow-up data were derived from the last contact with the institution and a minimum follow-up period of 24 months was required. If a patient had died or undergone revision surgery prior to the minimum follow-up, the patient was included in the final analysis. Early re-infections up to six weeks following prosthetic replacement were treated with debridement and component exchange while chronic infections underwent a further staged revision.

### Statistical analysis

All statistical analyses were conducted with Office Excel (Microsoft Corporation, Seattle, Washington, USA) and SPSS for Windows Version 26 (IBM Corporation, Armonk, New York, USA). Data distribution was analyzed using the Kolmogorov–Smirnov test and medians and IQR are reported. Parametric and non-parametric analyses were performed with the student’s *t* test or Mann–Whitney *U* test. Categorical variables were compared with the Chi-square test using cross-tabulation. Implant survivorship was analyzed using the Kaplan–Meier method and risk factors for implant failures were compared with the log-rank test reporting 95% CI. Factors that were found to have an influence on survivorship (*p* < 0.1) were included in a multivariate analysis using Cox regression analysis. The level of significance was set at *p* = 0.05 and all p values were two-sided.

A post hoc power analysis (G Power Version 3.1.9.7 [8]) (*χ* squared test) conducted for the rate of revision for reinfection revealed that a power of 0.99 could be achieved with the numbers available.

## Results

### Infection-free survivorship

**Fig. 2:**
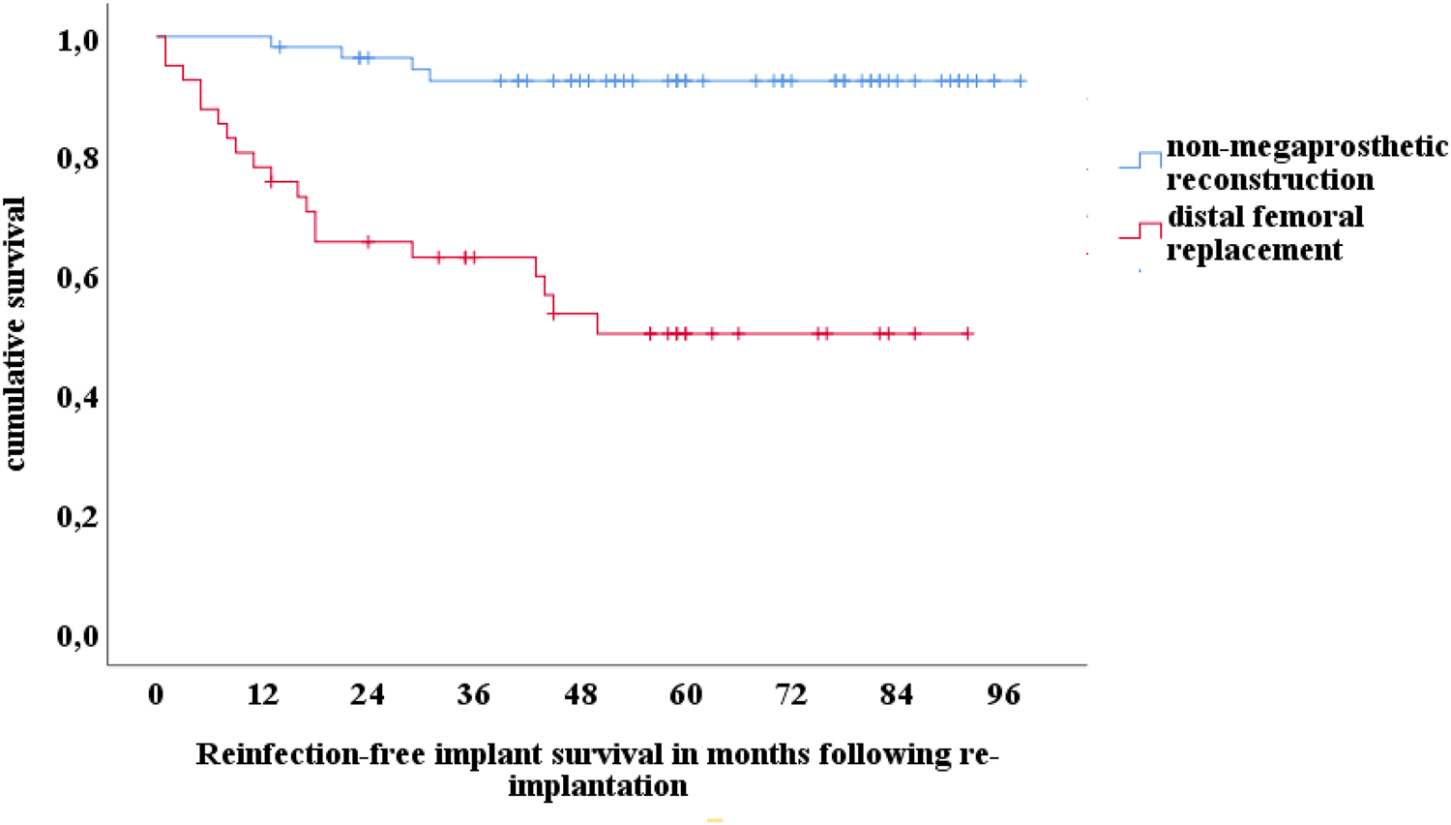
Kaplan–Meier implant survivorship curve for infection-free survival comparing distal femoral replacement and RHK revision TKA

In the entire cohort, 24% (23/97) of patients required revision surgery for infection. The infection-free survival was 83% (95% CI 76–90%) at two and 75% (66–84%) at five years. The survival of non-megaprosthetic revision TKA was 96% (95% CI 92–100%) and 93% (95% CI 86–100%) at two and five years, respectively. In this cohort, the reinfection rate was 7% (4/56). In contrast, in revision TKA with a DFR, the infection-free survivorship was 66% (95% CI 51–81%) and 50% (95% CI 34–66%), at two and five years, respectively (Fig. 2).

Of the 47% (19/41) DFR that underwent revision for reinfection, 26% (5/19) were retained after a debridement, irrigation and exchange of modular components for early or acute reinfection while 74% (14/19) had the prosthesis removed subsequently. The median time to a revision surgery for reinfection was 13 months (IQR 5–29) following second-stage reimplantation surgery.

Patients who underwent revision for reinfection had a higher BMI (34 (IQR 29–41) vs. 29 (IQR 26–33), *p* = 0.004), a longer median prosthesis length in mm (130 (IQR 105–195) vs. 90 (IQR 90–125, *p* = 0.009) and a higher median number of previous revision surgeries for infection (2 (IQR 0–3) vs. 0 (IQR 0–1), *p* = 0.003). On the other hand, the CCI (n.s.), a number of previous revision surgeries in general (n.s.) and the age at surgery (n.s.) were not associated with reinfection. Furthermore, patients with culture-negative or polymicrobial infection had not an increased risk for reinfection (n.s) (Tables 4 and 5). 22% (5/23) of patients who underwent revision for reinfection had the same organism at reinfection compared to the initial infection (Coagulase-negative *staphylococci* in all cases).

**Table 4:** microbiological results at first stage explantation

Organism	Distal femur % (*n*)	Non-mega-prosthetic % (*n*)
MSSA	22 (9/41)	9 (5/56)
(MR)- ConS	37 (15/41)	29 (22/56)
MRSA	5 (2/41)	none
Gram-negatives	2 (1/41)	9 (5/56)
Cutibacteria	2 (1/41)	4 (2/56)
VRE	2 (1/41)	2 (1/56)
Streptoccocus	2 (1/41)	2 (1/56)
others	5 (2/41)	9 (5/56)

*MSSA *methicillin sensitive* Staphylococcus aureus ConS *Coagulase-negative* Staphylococci MR-ConS *methicillin resistant Coagulase-negative* Staphylococci MRSA *methicillin resistant* Staphylococcus aureus VRE *vancomycin resistant* enterococci*

**Table 5:** microbiological results at reinfection

Organism	Distal femur replacement % (*n*)	Non-mega-prosthetic % (*n*)
MSSA	5 (1/19)	None
(MR)- ConS	57 (11/19)	75 (3/4)
MRSA	5 (1/19)	None
Gram-negatives	10 (2/19)	None
Cutibacteria	None	None
VRE	5 (1/19)	None
*Candida*	10 (2/19)	None
*Streptoccocus*	5 (1/19)	None
Others	None	50 (2/4)

*MSSA *methicillin sensitive* Staphylococcus aureus ConS *Coagulase-negative* Staphylococci MR-ConS *methicillin resistant Coagulase-negative* Staphylococci MRSA *methicillin resistant* Staphylococcus aureus VRE *Vancomycin resistant* enterococci*

Greater than 100% accounting for polymicrobial infections

In multivariate analysis, DFR was found to be at increased risk for reinfection compared to non-megaprosthetic reconstruction (HR 4.7 (95% CI 1–22), *p* = 0.048) as well as larger implants (HR 1.006 (95% CI 1.001–1.012), *p* = 0.032) and a higher BMI (HR 1.066, 95% CI 1.018–1.116), *p* = 0.007) while previous surgery for PJI was no risk factor (HR 1.165 (95% CI 0.371–3.662), *p* = 0.794).

### Amputation

While no patient with non-megaprosthetic reconstruction required amputation, there were 10% (4/41) above knee amputations in the group with DFR after a median period of 48 months (IQR 35–50) due to recurrent infections. This resulted in a probability of amputation of 5% (95% CI 2–8%) after five years and at final follow-up.

There were nine revision surgeries not related to infections. In the DFR cohort, there was one case of aseptic loosening of a cemented femoral stem and another case of aseptic tibial component loosening. Both underwent single stage revision. There was one case of wear of the coupling mechanism with inlay dislocation. In the non-DFR group, there were three cases of aseptic tibial loosening that underwent single stage revision and there were three soft tissue revision surgeries with exchange of the inlay and coupling mechanism. Of these, two were due to partial patellar tendon rupture that were reconstructed and augmented (in one case using a gastrocnemius flap) and one was due to chronic patella dislocation that was treated with medial reefing and lateral release. The median time to aseptic revision was 21 months (IQR 16–32).

## Discussion

The most important finding of this study was that DFR using a megaprosthesis was an independent risk factor for further revision surgery for infection particularly in patients with large defect reconstructions and high BMI. This subgroup of patients accounted to 10% of patients undergoing amputation after DFR, proving the hypothesis.

As the number of revisions and re-revision surgeries for PJI is expected to increase in the future [16], surgeons will be faced with the management of severe bone loss that might necessitate the use of megaprosthetic DFR [2, 14, 29]. Additionally, in selected cases, there might be borderline indications between augmented modular rotating hinge components and short segmental DFR. This might especially apply to subjects with poor bone quality as well as to elderly or comorbid patients in whom a shorter surgery time achieved by megaprosthetic reconstruction might be desirable. The results from this study contribute to the scant body of literature regarding DFR in the treatment of PJI and can aid surgeons in assessing the risk for further complications and patient consultation in complex revision TKA for PJI when megaprosthetic reconstruction is an considered an option.

Megaprosthetic reconstruction of the distal femur following revision surgery for PJI has been investigated in previous studies. Alvand et al. [1] included 29 implants and reported a failure rate due to infection in 41% of cases. These numbers are comparable to the current study. However, one must acknowledge that Alvand used antibiotic suppression therapy in 42% of recurring infections in the study. One might speculate that suppression therapy instead of further revision was offered to patients who were considered to be at high risk for further interventions or declined further surgery. In contrast, in the present study, all patients with recurrent PJI in the DFR group were advised further surgery and many underwent repeat two-stage exchanges or amputation following failed re-revisions. As these salvage procedures are all associated with a high mortality [19], antibiotic suppression might be an option to avoid further invasive procedures [2, 28]. However, this is only an option in cases where susceptible organisms are present that can be treated safely without severe adverse effects [20].

Wyles et al. [29] investigated 40 DFR reconstructions following two-stage exchange performed over a 15-year using four different megaprosthetic implant systems. 38% (15/40) of patients who were initially treated for PJI underwent further surgery due to infection, among those 10% (4/40) underwent amputation. The most common failure mode in that study was aseptic loosening, possibly due to the high percentage of aseptic indications for DFR. Considering the high reinfection rate found in the present study as well as in the study by Alvand et al., it seems that most patients who were treated for infections are more likely to develop recurring infections rather than aseptic loosening as the median infection-free survival was only 13 months in the present cohort.

Given the high probability of failures, the identification of risk factors for revision is important. In the present study, larger implants and patients with a higher BMI were at increased risk for repeated revision for infection. A longer reconstruction is associated with longer surgical time as well as greater soft tissue compromise, which might explain this finding. The length of a DFR was also identified as a risk factor for aseptic loosening by Wyles [29] finding a threefold risk for aseptic loosening and revision. However, in that study, the risk of infection has not been analyzed with respect to the implant size. In concurrence with the present findings, Barry et al. [2] identified longer DFR reconstructions reaching the diaphysis as a risk factor for infection in a cohort of 22 DFR that were performed over an 18-year period. The authors concluded that patients with a previous history of infection are at high risk for reinfection following DFR and consider these procedures final salvage treatments when other options to preserve a TKA have been exhausted. As other treatment options include amputation or fusion, it must be noted, that even after these procedures, notably high re-infection and revision rates have been reported [10, 15]. Hungerer et al. [15] found recurring infections in over 20% of all fusions when conducted with modular arthrodesis implants and in over 35% of all above-the-knee amputations.

Besides the high risk of reinfection for megaprosthetic DFR and rotating hinge knees, it was found that 4 patients had isolated tibial loosening at mid-term follow-up. While the most common approach for tibial fixation was a hybrid cementing technique with uncemented stems, the optimal stem fixation for revision TKA remains unknown [27]. While for revision after infection, the added antibiotics to the cement may have a positive effect on infection control, a stable fixation of a cemented stem usually requires inter-dentation with spongiosa that may not be present after prior stem exchange [27]. However, more recently porous metal cones have become quite popular showing excellent stability and survival [5, 21].

To interpret the findings of this study properly, some limitations must be considered. First, it is a retrospective study that is prone to selection and recall bias as it depends on follow-up data. Some patients might have undergone revision surgeries somewhere else. Consequently, the results presented must be considered low-end estimates of complications. Second, the mortality rate in the present study is quite high; death is a competing event when calculating implant survivorship resulting in a higher probability of failure using the Kaplan–Meier method. However, the mortality rate was not significantly different between the two study groups. A competing risk approach might result in a more appropriate estimate for survival, but nonetheless Kaplan–Meier survival probabilities are a relevant measure in consulting an individual patient [18] and ensures comparability to previous studies [29]. Lastly, some baseline demographics, such as the number of previous surgeries and previous PJI, are significantly different between patients undergoing DFR and non-megaprosthetic reconstruction. A multivariate approach including the number of previous surgeries to respect this difference in baseline demographics has been used. However, due to limited numbers available, there might be risk factors for failure that could not be accommodated in this study.

## Conclusion

Megaprosthetic DFR is a salvage treatment for infected (re-)revision TKA and is associated with an increased risk for reinfection compared to hinged non-megaprosthetic revision TKA.

Longer megaprosthetic reconstructions and patients with a higher BMI were at increased risk for further revision surgery. Patients must be counseled regarding these risks when DFR is planned or considered. To avoid further surgeries, suppression antibiotic therapy might be an option for selected patients that should be investigated in future studies.

## Abbreviations

BMI: Body mass index
CCI: Charlson comorbidity index
CI: Confidence interval
DFR: Distal femoral replacement
HR: Hazard ratio
IQR: Interquartile range 25–75% percentile
MSIS: Musculoskelettal Infection Society
MUTARS: Modular universal tumor and revision system
n.s.: non significant
PJI: Periprosthetic joint infection
PMMA: Polymethylmethacrylate
RHK: Rotating hinge knee
TKA: Total knee arthroplasty
